# Reversal of stage 5 chronic kidney disease by aortic valve replacement in kidney transplant recipient: a case report

**DOI:** 10.1186/s12872-020-01328-0

**Published:** 2020-01-17

**Authors:** E. Hryniewiecka, T. Hryniewiecki, J. Różański, T. Pilecki, R. Zagożdżon, T. Orłowski, M. Gołębiowski, L. Pączek, K. Mucha, B. Foroncewicz

**Affiliations:** 1 Department of Immunology, Transplantology and Internal Diseases, Medical University of Warsaw, 59 Nowogrodzka St, 02-006 Warsaw, Poland; 2 Department of Acquired Cardiac Defects, Institute of Cardiology, 42 Alpejska St, 04-628 Warsaw, Poland; 3 Department of Cardiosurgery and Transplantology, Institute of Cardiology, 42 Alpejska St, 04-628 Warsaw, Poland; 4 Department of Clinical Immunology, Medical University of Warsaw, 59 Nowogrodzka St, 02-006 Warsaw, Poland; 5 Department of Thoracic Surgery, National Tuberculosis and Lung Disease Research Institute, 26 Plocka St, 01-138 Warsaw, Poland; 6 Department of Clinical Radiology, Medical University of Warsaw, 59 Nowogrodzka St, 02-006 Warsaw, Poland; 7 Institute of Biochemistry and Biophysics, Polish Academy of Sciences, 5A Pawinskiego St, 02-106 Warsaw, Poland

**Keywords:** Kidney transplantation, End stage renal disease, Acute-on-chronic, Haemodialysis, Cardiorenal syndrome, Aortic insufficiency, Heart failure, Aortic valve replacement

## Abstract

**Background:**

Cardiorenal syndrome (CRS) is a group of pathophysiological disorders affecting heart and kidneys.

**Case presentation:**

We present 44-year-old kidney transplant recipient with acute-on-chronic graft failure in the course of CRS due to acutely decompensated heart failure associated with severe aortic regurgitation successfully treated with aortic valve replacement. Because of graft failure progression and difficult to eradicate infections he was treated with dialysis and radical minimization of immunosuppression. After 74 days of renal replacement therapy the patient regained graft function after successful aortic valve replacement. The dialysis could be stopped and immunosuppressive therapy was reintroduced. Heart and renal function are stable and patient is doing well without dialysis for 3 years.

**Conclusions:**

The return of kidney graft function can occur even after a long period of dialysis therapy due to improved cardiovascular function. Therefore, distinguishing an acute-on-chronic CRS subtype is mandatory to enable specific patient approach.

## Background

Aortic regurgitation (AR) leads to diastolic retrograde flow of blood from the aorta to the left ventricle (LV). It results in LV volume overload, dilatation and dysfunction. High regurgitant volume lowers diastolic pressure and causes decreased end organ perfusion. Heart failure (HF) and inadequate peripheral perfusion deteriorate function of chronically impaired kidneys. For the first time cardiorenal syndrome (CRS) was described in 1951 [1]. At present, it is considered a group of pathophysiological disorders affecting heart and kidneys. Depending on the timing and sequence of events CRS is classified into: type 1 acute CRS (rapid worsening of cardiac function induces acute kidney injury), type 2 chronic CRS (chronic HF induces progressive renal injury), type 3 acute renocardiac syndrome (rapid worsening of kidney function induces acute HF), type 4 chronic renocardiac syndrome (chronic renal impairment induces progressive HF), and type 5 CRS (systemic disease causes simultaneous dysfunction of the heart and kidneys) [2]. Scientific interest in CRS affecting native kidneys is increasing whereas post-kidney-transplant CRS data is very scarce and outcomes of this condition are sought [3]. In kidney transplant recipients the frequency of graft loss by CRS is estimated 5% [3].

We present kidney transplant recipient with CRS due to acutely decompensated HF associated with severe AR successfully treated with aortic valve replacement (AVR). The patient regained his kidney graft function after 74 days of chronic hemodialysis and radical minimization of immunosuppression and he continues without dialysis for 3 years.

## Case presentation

The medical history of 44-year old male kidney transplant recipient with chronic allograft nephropathy started in 1994 with arterial hypertension and reflux nephropathy causing end stage kidney disease (ESKD) and bilateral nephrectomy for infected hydronephrosis. He has been hemodialyzed for 6 years before he was transplanted in July 1999. His post-transplant immunosuppression consisted of steroids, cyclosporine and azathioprine, which was converted to mycophenolate mofetil (MMF) in March 2000 after treatment of Banff IA rejection with pulses of methylprednisolone. Immunosuppressive therapy was complicated with cytomegalovirus infection (October 2000); fungal encephalitis (antifungal therapy from October 2008 to February 2009); Epstein-Barr virus-positive Hodgkin lymphoma IIIA treated with 8 courses of rituximab and 6 courses of doxorubicin, bleomycin, vinblastine and dacarbazine (January – October 2014) and gancyclovir-resistant cytomegalovirus reactivation treated with conversion from MMF to leflunomide (October 2014 – February 2015). Because of active and chronic antibody-mediated rejection (AMR) and chronic transplant glomerulopathy diagnosed on biopsy (May 2015) leading to progression of graft insufficiency leflunomide was withdrawn and the patient continued cyclosporine 25 mg twice daily (BID) with through levels 33.68–56.6 ng/ml and prednisone 5 mg once daily (QD).

On April 2015 the patient presented with fever, malaise, increased intensity of systolic murmur and elevated C-reactive protein (CRP). Echocardiography revealed aortic valve (AV) disease with predominant severe AR of unknown duration, preserved ejection fraction (EF = 65%) and hyperkinetic myocardium with pulmonary hypertension. There were no obvious signs of endocardial vegetations, nor positive blood cultures. The patient was diagnosed with staphylococcal sepsis, cytomegalovirus (CMV) reactivation, and labial herpes simplex and was treated with ceftriaxone, clindamycin, vancomycin, acyclovir and fluconazole with clinical and laboratory improvement. In June 2015 he was qualified for aortic surgery as the treatment of mixed aortic valve disease (severe aortic regurgitation and moderate aortic stenosis).

In October 2015 while on a waiting list, he was admitted to the tertiary hospital because of aggravating biventricular HF with orthopnea and sub-febrile temperature. The diagnosis of bilateral pneumonia was established and AV infective endocarditis (IE) was suspected. Antimicrobial therapy (ciprofloxacin, ceftriaxone, fluconazole) and hemodialysis because of acute-on-chronic graft failure were initiated. The patient was referred to our department after one week of treatment.

On examination, he was found to be in class IV of heart failure according to New York Heart Association (NYHA), tachycardia 100–120/min, grade 5 continuous murmur, dyspnea requiring oxygen therapy, crackles in the lower half of the lung fields, prominent peripheral edema and partial edentulism.

**Fig. 1:**
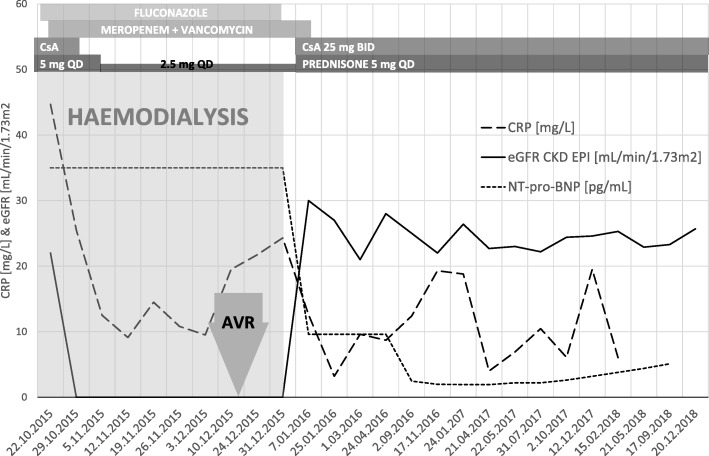
Time course of changes of main laboratory parameters along with administered therapy. AVR, aortic valve replacement surgery; CRP, C-reactive protein; CsA, cyclosporine; eGFR_cr_ CKD EPI, estimated glomerular filtration rate by Chronic Kidney Disease Epidemiology Collaboration equation; BID, two times a day; QD, one a day

The laboratory tests revealed leukocytosis, anemia, 4 stage chronic kidney disease (CKD), and elevated total bilirubin (TB), CRP and N-terminal pro B-type natriuretic peptide (NT-pro-BNP) (Fig. 1). Echocardiography showed fibrosis and calcifications of AV with mobile echogenic mass attached, prominent AR and mild stenosis, moderate enlargement of LV with mild hypokinesis, EF = 50%, and increased right ventricle systolic pressure. Abdominal ultrasound revealed enlarged spleen (151 mm), mild bilateral pleural and peritoneal effusion, increased intrarenal artery resistive index with retrograde diastolic blood flow in graft artery and decreased flow in graft vein. Computed tomography (CT) revealed enlarged cardiac silhouette, moderate pulmonary circulation overload and round lesion in the upper right pulmonary field (30 mm) suggesting pulmonary abscess. Bronchoscopy revealed acute purulent bronchitis and caused by *Staphylococcus haemolyticus* susceptible to vancomycin only. All blood cultures were negative for bacteria and fungi. Based on the above findings, the following diagnoses were established: bilateral lobar pneumonia with infected emphysematous bulla of the right lung, culture-negative aortic valve IE, AV disease with severe AR and biventricular HF decompensation overlapping with chronic graft nephropathy with kidney insufficiency requiring hemodialysis.

**Fig. 2:**
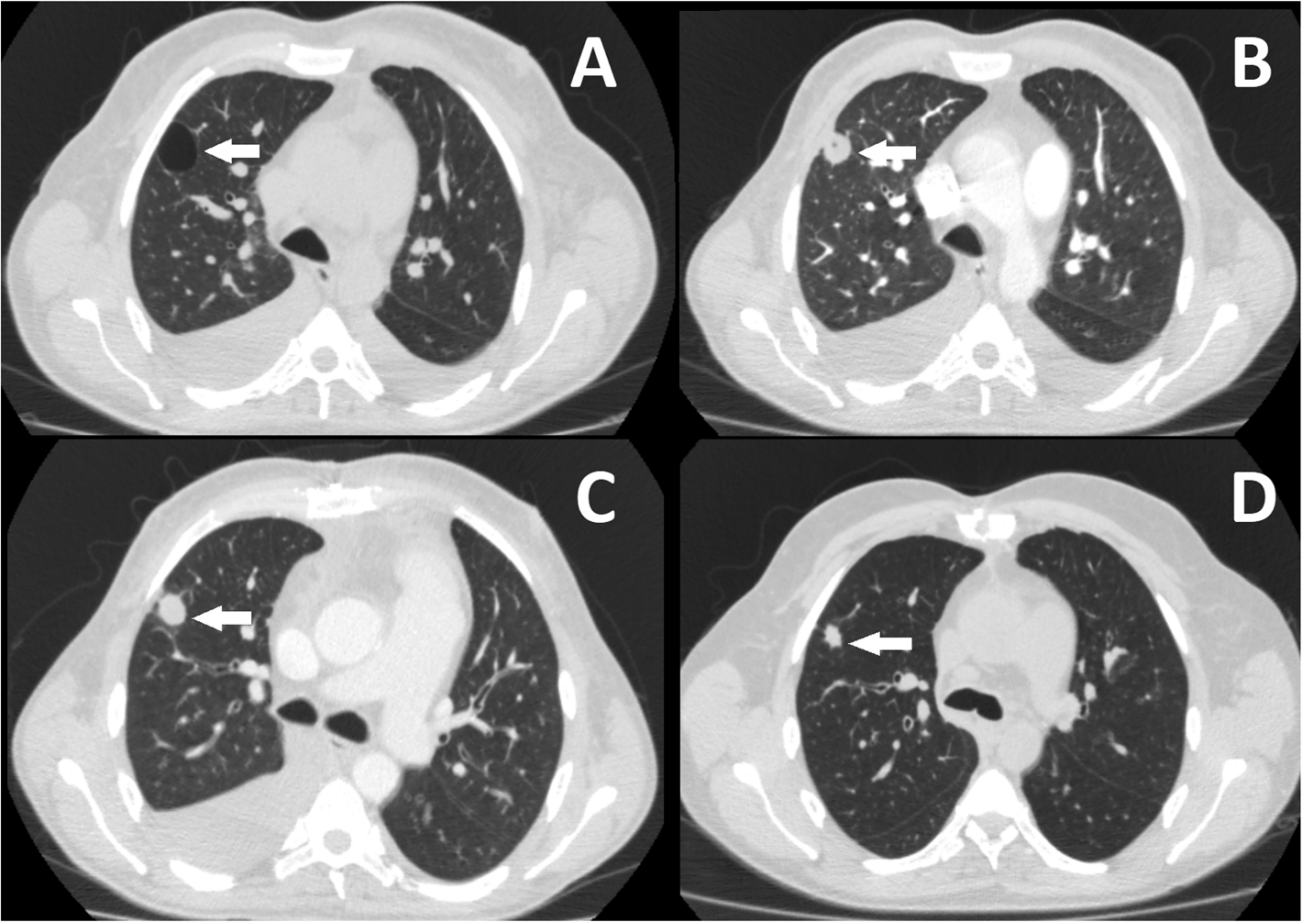
Evolution of pulmonary lesions over time by high resolution computed tomography. **a** – 20-01-2014; **b** – 6-11-2015; **c** – 29-12-2015; **d** – 15-04-2016. The white arrow indicates localization of pulmonary abscess

The patient was administered meropenem, vancomycin and fluconazole. Because of clinical features suggesting ESKD daily hemodialysis was continued, cyclosporine was discontinued and prednisone was tapered (Fig. 1). After approximately 3 weeks CT revealed partial regression of pulmonary consolidations and reduced diameter of infected bulla (Fig. 2b). No significant stenosis was found on coronary angiography. Therefore, in the context of hemodynamic stabilization and normalization of inflammatory parameters due to significant destruction of AV leaflets the tissue valve Medtronic Hancock II 27 mm was implanted on the 16th December 2015. Surgery revealed the presence of two healed penetrating lesions possibly associated with IE. The native valve cultures were negative.

15 days postoperatively hemodialysis (total duration of 74 days from 18.10.2015 to 31.12.2015) was withheld because of increasing diuresis and improvement of graft function. Minimized immunosuppressive therapy (prednisone 5 mg QD and cyclosporine 25 mg BID) was readministered. At the beginning of January 2016, the results of additional tests revealed: eGFR 32 mL/min/1.73 m2 with substantial decrease of TB, CRP and NT-pro-BNP with NYHA class reduction (II) (Fig. 1). Chest CT showed further regression of pulmonary consolidations and reduction of infected bulla (Fig. 2c) and patient was discharged home on the 11th January 2016.

Three months later bilateral lobar pneumonia recurred followed by deterioration of kidney function. It was successfully treated with meropenem and vancomycin. At the end of therapy, the eGFR_cr_ was 28 mL/min/1.73 m2, the CT shown further regression of infected bulla (Fig. 2d) and two consecutive echocardiographies have revealed good function of AV prosthesis with mean/maximal transvalvular gradient of 19/34 mmHg and 16/39 mmHg and EF = 50 and 62%. At present 3 years after AVR, the patient maintains graft function (estimated glomerular filtration rate (eGFR_cr_) 22.2 mL/min/1.73 m2) while on prednisone 5 mg QD and cyclosporine 25 mg BID (trough levels = 29.03–48.1 ng/mL).

## Discussion and conclusions

To the best of our knowledge, this is the first report of return from chronic (74 days) hemodialysis after successful CRS treatment with AVR. Kim and Lee described a case of 82-year-old man with decompensated heart failure due to severe aortic stenosis, which was successfully treated with emergency transcatheter aortic valve replacement [4]. The described patient did not required dialysis despite administering radiographic contrast. In the literature we did find one similar case of presumed CRS resolved after AVR due to aortic insufficiency caused by IE [5]. However, there are some essential differences between these two cases. The duration of graft failure (not requiring renal replacement therapy) in the case described by Masmoudi et al. was unknown. In our patient stage 5 CKD lasted over two months suggesting chronic irreversible ESKD (two weeks were missing to meet the 3 months criterion for ESKD). Additionally, more comorbidities and chemotherapy adversely affected renal function in our patient. The valve types were also different (Hancock II vs St Jude Medical). The main difference between these cases is the absence of the need for dialysis and very rapid kidney graft function improvement after AVR surgery. Another cause of CRS was reported by Nickel et al. A kidney transplant recipient with HF due to high flow arteriovenous fistula leading to deterioration of graft function which improved after operative flow reduction [6]. Also, in this case the patient did not require renal replacement therapy and immunosuppressive therapy was continued all the time.

A prerequisite for the safe surgery for decompensated AV disease was the treatment of infected bulla and IE. It was facilitated by the discontinuation of immunosuppressive therapy, which was no longer necessary because of graft insufficiency and hemodialysis. Only after the cardiac surgery it became clear that CRS had been the main cause of graft dysfunction. Importantly, regain of graft function after operation was unexpected. Over two months of hemodialysis in patient with chronic progressive kidney disease practically precluded his chances for nephrological improvement. A case of native kidneys’ function recovery after 8 years of renal replacement with kidney graft in patient with membranous nephropathy was described [7]. Due to accidental detection of renal function recovery the authors were not able to determine exact duration of ESKD. In addition, in that case native renal function recovered most probably as a result of intensive immunosuppression after kidney transplantation, and not as in our case, despite discontinuation of such a treatment. Therefore, this novel observation suggests the need for prolonged thorough kidney function follow-up in patients with CRS on hemodialysis. Moreover, given the clinical course of CRS in our patient we emphasize the need to distinguish a specific type of CRS1, i.e. acute HF inducing acute-on-chronic kidney disease. In classic CRS1 the acute renal failure suggests the possibility of its reversal. In contrast, the chances to revert the acute-on-chronic renal failure, like in our patient are much lower, particularly in advanced CKD stages.

In conclusion, AVR is feasible and effective therapy even in high-risk patients with severe infections and comorbidities, and chronic hemodialysis-dependent stage 5 CKD may be reversible. We emphasize the need to distinguish a subtype of CRS1 for patients with acute-on-chronic renal failure due to acute heart insufficiency. If hemodialysis is needed in such patients, prolonged renal function follow-up is required after cardiac therapy. The main and extremely important contribution to the medical knowledge of the presented case report is demonstration of: 1) restoring advanced renal failure after nearly three months of hemodialysis, 2) possibility of treating of advanced and chronic graft kidney disease by valve replacement, 3) not described in the literature so far possibility of cardiac valve surgery in patient with significant comorbidity and after kidney transplantation, 4) the need for specific, personalized and interdisciplinary approach to the patient with CRS1, 5) the need for prolonged renal function follow-up in patients with CRS after cardiac surgery.

## Data Availability

Not applicable.

## Abbreviations

AMR: Antibody-mediated rejection
AR: Aortic regurgitation
AV: Aortic valve
AVR: Aortic valve replacement
BID: Twice daily
CKD: Chronic kidney disease
CMV: Cytomegalovirus
CRP: C-reactive protein
CRS: Cardiorenal syndrome
CT: Computed tomography
EF: Ejection fraction
eGFR_cr_: Estimated glomerular filtration rate
ESRD: End stage renal disease
HF: Heart failure
IE: Infective endocarditis
LV: Left ventricle
MMF: Mycophenolate mofetil
NT-pro-BNP: N-terminal pro B-type natriuretic peptide
NYHA: New York heart association
QD: Once daily
TB: Total bilirubin
